# Sex-related differences in lung injury distribution and outcomes in COVID-19 acute respiratory failure: insights from the CT-COVID19 multicenter study group

**DOI:** 10.1186/s40635-026-00914-4

**Published:** 2026-06-16

**Authors:** Davide Signori, Alice Nova, Yi Xin, Sarah E. Gerard, Aurora Magliocca, Giovanni Vitale, Linda Mussoni, Jonathan Montomoli, Matteo Subert, Alessandra Ponti, Savino Spadaro, Giancarla Poli, Francesco Casola, Roberta Garberi, Andrea Restivo, Davide Raimondi Cominesi, Marco Giani, Vanessa Zambelli, Giuseppe Foti, John G. Laffey, Maurizio Cereda, Emanuele Rezoagli

**Affiliations:** 1 Department of Anesthesia and Intensive Care, Azienda Socio Sanitaria Territoriale Bergamo Est, Seriate, Italy; 2 School of Medicine and Surgery, University of Milano-Bicocca, Monza, Italy; 3 Department of Emergency and Intensive Care, Fondazione IRCCS San Gerardo Dei Tintori Hospital, Monza, Italy; 4 Department of Anesthesiology, Critical Care, and Pain Medicine, Massachusetts General Hospital, Harvard Medical School, Boston, MA USA; 5 Department of Anesthesiology and Critical Care, University of Pennsylvania, Philadelphia, USA; 6 Roy J. Carver Department of Biomedical Engineering, University of Iowa, Iowa City, Iowa USA; 7 Department of Anesthesia and Intensive Care Medicine, Policlinico San Marco, Gruppo Ospedaliero San Donato, Bergamo, Italy; 8 Department of Pathophysiology and Transplantation, University of Milan, Milan, Italy; 9 Istituto per la Sicurezza Sociale, San Marino, San Marino, Italy; 10 Department of Anesthesia and Intensive Care, Infermi Hospital, AUSL Romagna, Rimini, Italy; 11 Health Services Research, Evaluation and Policy Unit, Romagna Local Health Authority, Rimini, Italy; 12 Department of Anesthesia and Intensive Care Medicine, Melzo-Gorgonzola Hospital, Azienda Socio-Sanitaria Territoriale Melegnano e della Martesana, Melegnano, Milan Italy; 13 Department of Anesthesia and Intensive Care Medicine, Azienda Ospedaliero-Universitaria of Ferrara, Ferrara, Italy; 14 Department of Translational Medicine, University of Ferrara, Ferrara, Italy; 15 Department of Anaesthesia and Critical Care Medicine, Papa Giovanni XXIII Hospital, Bergamo, Italy; 16 Department of Physics, Harvard University, Cambridge, MA USA; 17 Harvard-Smithsonian Centre for Astrophysics, Cambridge, MA USA; 18 School of Medicine, National University of Ireland Galway, Galway, Ireland; 19 Department of Anaesthesia and Intensive Care Medicine, Galway University Hospitals, Galway, Ireland

**Keywords:** Computed tomography, Superimposed pressure, Sex differences, COVID-19, Acute hypoxemic respiratory failure, Mortality

## Abstract

**Background:**

Sex-related differences have been consistently reported in the epidemiology of acute hypoxemic respiratory failure (AHRF) and COVID-19. However, whether computed tomography (CT)-derived measures of lung injury differ between sexes and contribute to outcome disparities remains unclear.

**Methods:**

In this large multicenter retrospective cohort study, we analyzed 850 spontaneously breathing patients with COVID-19-related AHRF who underwent early chest CT at hospital admission. Quantitative CT analysis provided measures of lung density, volume, mass, and superimposed pressure (SP), a CT-derived estimate of gravitational stress. Sex-stratified analyses compared morphological, physiological, and outcome variables. Multivariable logistic regression models identified independent predictors of mortality.

**Results:**

Among 850 patients (35% women), men exhibited larger lung volume (2.91 vs. 2.28 L, *p* < 0.001), greater lung mass (1.14 vs. 0.93 kg, *p* < 0.001), and higher SP (5.79 vs. 5.21 cmH₂O, *p* < 0.001) despite similar fractions of ground-glass opacities and consolidation. In the multivariable model, older age (OR 1.08, 95% CI 1.06–1.11; *p* < 0.001), lower PaO_2_/FiO_2_ (OR 0.99, 95% CI 0.98–0.99; *p* < 0.001), higher SOFA score (OR 2.67, 95% CI 1.43–4.98; *p* = 0.002 for SOFA ≥ 2), higher global SP (OR 1.18, 95% CI 1.05–1.34; *p* = 0.005), and male sex (OR 1.76, 95% CI 1.06–2.92; *p* = 0.028) were independently associated with an increased risk of mortality. In the mediation analysis, the effect of global SP on mortality does not appear to be mediated by male sex (coefficient 0.00).

**Conclusions:**

Male patients with COVID-19-related AHRF exhibited higher global SP than females, reflecting greater gravitational lung load and mechanical disadvantage. Both global SP and male sex were independently associated with mortality, with no evidence of mediation of male sex on mortality. These finding suggest that, beyond anatomical and mechanical differences, biological and hormonal factors likely contribute to the increased disease severity observed in men.

**Supplementary Information:**

The online version contains supplementary material available at 10.1186/s40635-026-00914-4.

## Introduction

In acute hypoxemic respiratory failure (AHRF) and its more severe form, acute respiratory distress syndrome (ARDS), chest computed tomography (CT) has played a pivotal role in understanding pathophysiology [1, 2], leading to the conceptualization of the “baby lung”[3] and “sponge lung” [4] models.

Today, CT continues to be used in clinical practice to assess disease severity and treatment response, suggest potential underlying etiologies, and identify complications, such as pleural effusion, empyema, barotrauma, or pulmonary embolism [5]. For research purpose, CT provides both quantitative [6] and qualitative [7] evaluations of lung injury severity. Among quantitative indices, the superimposed pressure (SP), the vertical pressure gradient exerted by the weight of the overlying lung tissue, has been proposed as a CT-derived marker of lung collapse and gravitational stress, providing a functional correlate of parenchymal inhomogeneity and mechanical load [8, 9].

More recently, CT imaging has contributed to the identification of distinct subgroups of critically ill patients within otherwise heterogeneous populations [10, 11]. In patients with ARDS [12], CT scan has demonstrated its potential to improve prognostication [13] and to guide individualized therapeutic strategies [14]. Furthermore, incorporating CT analysis into AHRF phenotyping [15, 16] appears to be a valuable approach for advancing precision medicine [17, 18]. In a large multicenter dataset of spontaneously breathing patients with COVID-19-related AHRF, we demonstrated that integrating quantitative CT features with clinical parameters could identify two distinct phenotypes, which were differently associated with hospital mortality after adjustment for clinically meaningful variables [19].

Beyond morphological heterogeneity, patient-related factors such as biological sex may further modulate the severity and outcomes of lung injury. Evidence from large international cohorts indicates that sex can influence outcomes in ARDS. In a secondary analysis [20] of the LUNG SAFE study [21], female sex was independently associated with lower intensive care unit (ICU) mortality after adjustment for severity and comorbidities, suggesting a potential biological or physiological basis for sex-specific differences in ARDS outcomes. Sex-related differences in critical illness have received increasing attention during the COVID-19 pandemic. Men consistently exhibited higher risk of hospitalization, AHRF severity, and mortality compared with women [22, 23]. Biological mechanisms—including hormonal regulation, immune responses, and cardiovascular risk profiles—may contribute to this disparity [24–27].

Although the influence of sex on outcomes in critical illness is well-established, and CT imaging plays a central role in characterizing lung injury, it remains unknown whether imaging-based markers of lung injury differ by sex and whether such differences could partly explain outcome variability between men and women.

We hypothesized that quantitative CT-derived measurements would differ between male and female patients with AHRF, and that sex-related differences in distribution patterns of lung injury might contribute to disparities in outcomes between men and women. Our primary aim is to assess whether CT-derived features of COVID-19-related AHRF differ by sex. Second, we aim to evaluate whether SP contributes to outcome disparities between sexes.

## Materials and methods

### Ethical consideration and data acquisition

The study was conducted in accordance with the Declaration of Helsinki and the Italian Good Clinical Practice recommendations (D.M. Sanità 15/07/97 and subsequent amendments), as well as with the approved healthcare protocols of the participating hospitals, as previously described [19]. Baseline characteristics (age, sex, body mass index, comorbidities) and clinical illness severity score (Sequential Organ Failure Assessment—SOFA) were collected, together with laboratory biomarkers, blood gas analysis, respiratory assistance, and hemodynamic data at hospital admission. Chest CT scans performed for clinical purposes within the first week after hospital admission were obtained. Data on clinical course and in-hospital complications, need for respiratory support, limitation of life sustaining measures, and clinical outcome were acquired. Clinical, laboratory and CT data were collected between February and April 2020, during the peak of the first Italian wave of the COVID-19 pandemic. Details about ethical considerations and data acquisition are reported in the supplementary materials.

### Inclusion and exclusion criteria

#### Inclusion criteria:

1. Patients ≥ 18 years;
2. Positive confirmation of SARS-CoV-2 infection with nucleic acid amplification test or serology of SARS-CoV2 by nasopharyngeal swab, broncho-aspirate sample or bronchoalveolar lavage;
3. Clinical diagnosis of respiratory faiulure at Emergency Department admission;
4. Lung CT scan performed within 7 days since hospital admission.

All patients included in the current analysis were admitted to the Emergency Department with a clinical diagnosis of respiratory failure.

#### Exclusion criteria:

1. Patients undergoing mechanical ventilation during CT acquisition;
2. Patients with CT data that cannot be used to estimate CT derived parameters, such as SP (e.g., low-resolution images or scans not including the entire lung).

### Chest CT quantification

All lung CT images were collected, anonymized, and transferred via the University of Milano-Bicocca Institutional Google drive account to Massachusetts General Hospital, Boston, MA, USA (M.C., Y.X.) and The University of Iowa, Iowa City, IA, USA (S.G) in a fully de-identified format for advanced quantitative analysis. Advanced quantitative analysis was performed using artificial intelligence with deep learning algorithms [28]. Specifically, CT images were segmented using a previously validated convolutional neural network (CNN) [29, 30]. The segmentation masks included pulmonary vasculature and intrapulmonary airways, while major airways (e.g., trachea) and hilar vessels outside the lung lobes were excluded. The CNN automatically labeled three regions of interest (ROIs) for subsequent analysis: the whole lung, left lung, right lung. Each lung ROI was further divided into four quadrants, referred as Left Basal (LB), Left Apical (LA), Right Basal (RB), and Right Apical (RA). These quadrants were defined by dividing the lung into equal segments along the basal-to-apical axis. After segmentation, whole-lung and lobar masks were inspected by trained investigators (Y.X. and A.N.), and manually adjusted using ITK–SNAP software [31]. For each ROI, ten quantitative parameters were analyzed [19]. The quantitative parameters and their technical specification are reported below:

- Lung density (HU): average CT density in Hounsfield Units (HU)
- Lung volume (L): pixel count × pixel dimensions
- Lung gas volume (L): lung volume × (HU/− 1000)
- Lung mass (Kg): lung volume × (1 – HU/− 1000)
- Consolidation fraction (%): CT density $\ge$ −200 HU [32, 33]
- GGO fraction (%):−200 HU > CT density $\ge$ −750 HU [34]
- Total injury fraction (%): CT density $\ge$ −750 HU
- Lung height (cm): maximum dorso-ventral height
- Global SP (cmH_2_O): average CT density × maximum ventro-dorsal lung height [35]
- Regional SP (cmH_2_O):
  - Ventral-to-dorsal regional SP: calculated by dividing the lung into 10 equally spaced, gravitationally distributed sections (each representing one-tenth of the maximum dorso-ventral lung height). For each section, SP was obtained as the product of the CT density and the section’s dorso-ventral height, cumulatively adding the SP of the sections above [1].
  - Basal-to-apical regional SP: calculated by dividing the lung into 10 equally spaced, craniocaudally distributed sections (each representing one-tenth of the maximum apico-basal height). For each section, SP was obtained as the product of the CT density and the corresponding ventro-dorsal height [36].

### Statistical analysis

Continuous data are reported as mean ± standard deviation (SD) or median and interquartile range (IQR). Categorical variables are expressed as proportions (frequency). Differences in continuous data were assessed by *T* test or Mann–Whitney test, as appropriate. Differences in categorical data were assessed by chi-square test.

Correlations between quantitative lung computed tomography data and gas exchange were assessed by Pearson linear correlation coefficient. The correlations between the qualitative and quantitative variables were obtained using univariable and multivariable logistic regression. Variables showing a significant association with the outcome in univariable analysis (*p* < 0.05) (Table S1) and considered clinically relevant based on prior literature and biological plausibility were included. In cases where multiple candidate predictors reflected overlapping biological or physiological mechanisms or were highly correlated, only one variable was selected for inclusion in the multivariable model to avoid redundancy and collinearity. Collinearity among candidate predictors was assessed using variance inflation factors (VIF). An unadjusted Kaplan–Meier survival analysis at the 90-day follow-up was performed to determine the probability of survival in male and female patients. Differences between the stratified populations was assessed using the log-rank test.

Statistical significance was considered with a *p* < 0.05 (two-tailed). Statistical analysis was performed by using Stata/MP version 17.0 for Mac (Copyright 1985–2021, StataCorp LLC, College Station, TX, USA) and GraphPad Prism version 10.2.2 (Copyright 1994–2003, GraphPad Software, Inc., San Diego, California).

## Results

### Study population

A total of 853 patients were initially screened, of whom 850 patients met inclusion criteria: 296 women (35%) and 554 men (65%). The time between hospital admission and CT scan was 0 days [0–0] in women and 0 [0–1] in men (*p* = 0.138). Baseline demographic characteristics, comorbidities, clinical illness severity and respiratory support at hospital admission, as well as clinical outcome stratified by sex are summarized in Table S1.

Median body mass index did not differ between sexes (27.3 [24.3–31.2] kg/m^2^ in women vs. 27.6 [24.7–30.1] kg/m^2^ in men, *p* = 0.635). The overall prevalence of comorbidities was similar (64% in women vs. 66% in men, *p* = 0.584). However, women more often had immune-mediated diseases (6% vs. 2%, *p* = 0.010) and thyroid dysfunction (11% vs. 3.5%, *p* < 0.001).

Men presented with higher lymphocytes counts (6.7[4.9; 9.3] vs. 6.5[4.9;9.0], p = 0.021) and slightly higher body temperature (37.7 °C vs. 37.5 °C, *p* = 0.002). Women had higher platelet counts (204 vs. 180 × 10^3^/µL, *p* < 0.001). Men showed higher levels of creatinine (1.12 vs. 0.87 mg/dL, *p* < 0.001), urea (44 vs. 34 mg/dL, *p* < 0.001), and CPK (149 vs. 103 UI/L, *p* = 0.001), as well as higher bilirubin (0.61 vs. 0.47 mg/dL, *p* < 0.001), and transaminases levels (AST and ALT, both *p* ≤ 0.006).

Most patients were spontaneously breathing on room air at presentation, and respiratory support use and modality did not significantly differ between men and women (p = 0.095). Arterial blood gas analysis revealed more severe hypoxemia in men (PaO_2_/FiO_2_ 255 [145–315] mmHg vs. 282 [187–340] mmHg in women, *p* < 0.001), whereas admission pH was similar between sexes (7.47 [7.43; 7.49] in women vs. 7.47 [7.44; 7–49] in men, *p* = 0.761).

Bacterial over-infection was similar between men and women (12% vs. 16%, *p* = 0.141), while the incidence of pneumothorax showed a trend toward being higher in men (0.3% vs. 2%, *p* = 0.097). Venous thromboembolism occurred more frequently in men (3% vs. 1%, *p* = 0.045).

### CT quantitative analysis

Quantitative CT metrics stratified by sex are reported in Table S2.

#### Whole lung analysis

**Fig. 1:**
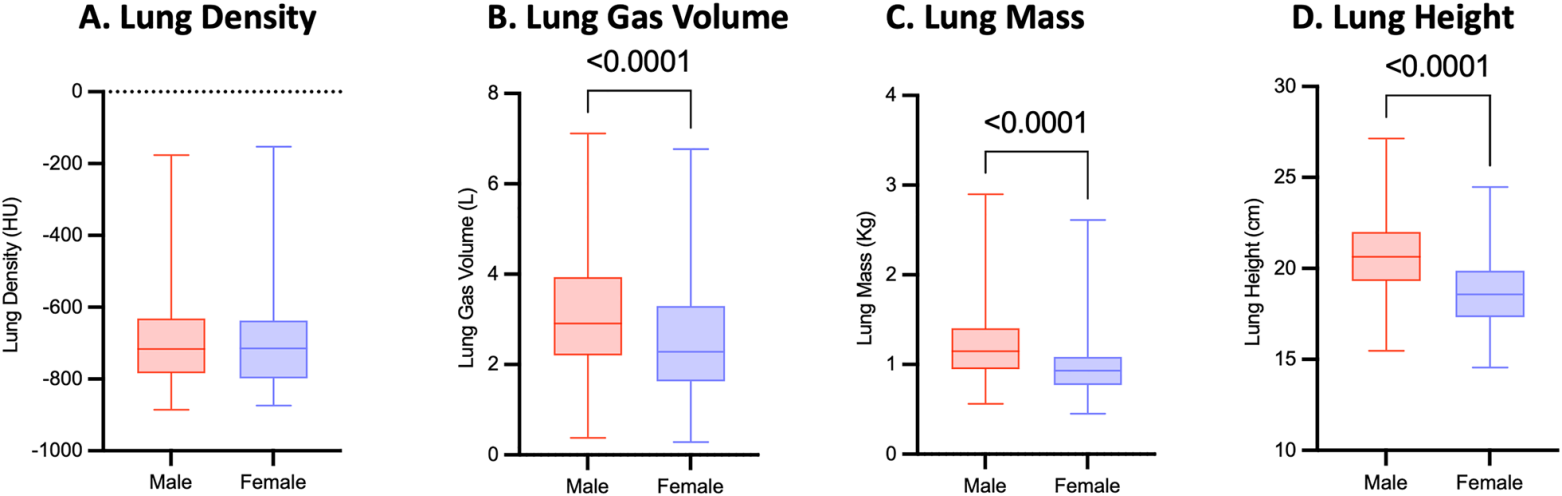
Lung Density (**A**), Lung Gas Volume (**B**), Lung Mass (**C**), and Lung Height (**D**) in male and female patients. *N* male = 554; *N* female = 296; data are presented as box and whiskers (min. to max., line at median)

**Fig. 2:**
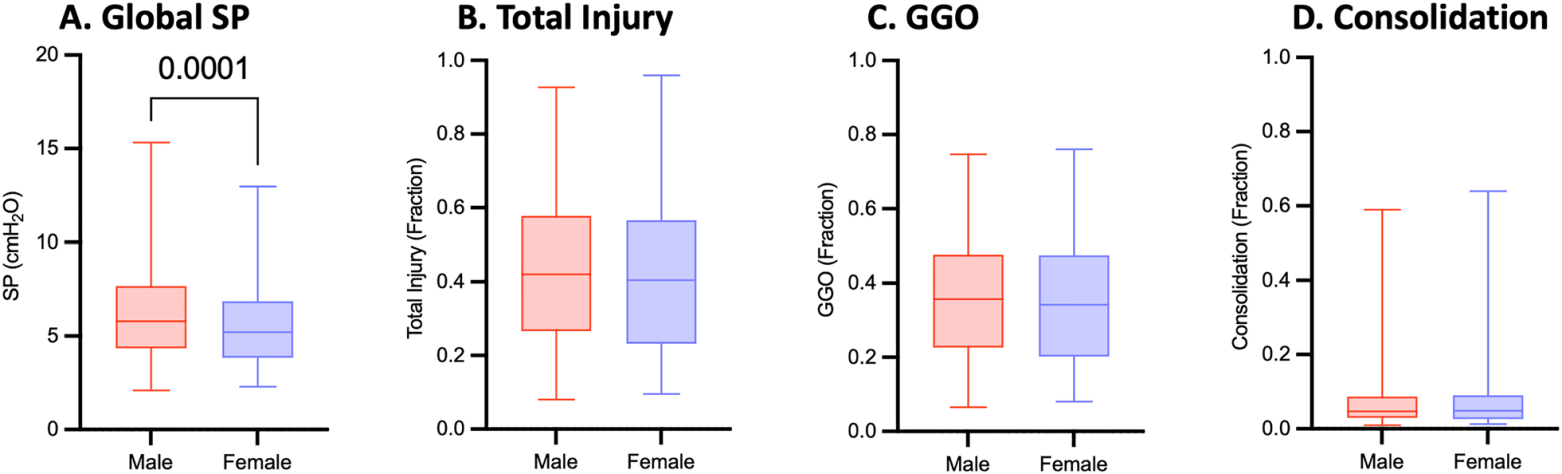
Global Superimposed Pressure (**A**), Total Injury Fraction (**B**), GGO Fraction (**C**), and Consolidation Fraction (**D**) in male and female patients. *N* male = 554; *N* female = 296; data are presented as box and whiskers (min. to max., line at median). GGO: ground glass opacities; SP: Superimposed Pressure

Global lung density did not differ significantly between sexes (−715 HU in women vs. −716 HU in men, *p* = 0.691). However, men showed consistently larger lung gas volumes (2.91 [2.21–3.94] vs. 2.28 [1.63–3.29] L, *p* < 0.001), greater lung mass (1.14 [0.95–1.41] vs. 0.93 [0.77–1.08] kg, *p* < 0.001), and increased lung height (20.66 ± 1.90 vs. 19.70 ± 1.93 cm, *p* < 0.001) (Fig. 1). Global SP was also significantly higher in men (5.79 [4.35–7.67] vs. 5.21 [3.86–6.84] cmH_2_O, *p* < 0.001). In contrast, the fraction of total lung injury, ground-glass opacities, and consolidation were similar across sexes (all *p* > 0.2; Fig. 2).

#### Spatial injury distribution.

**Fig. 3:**
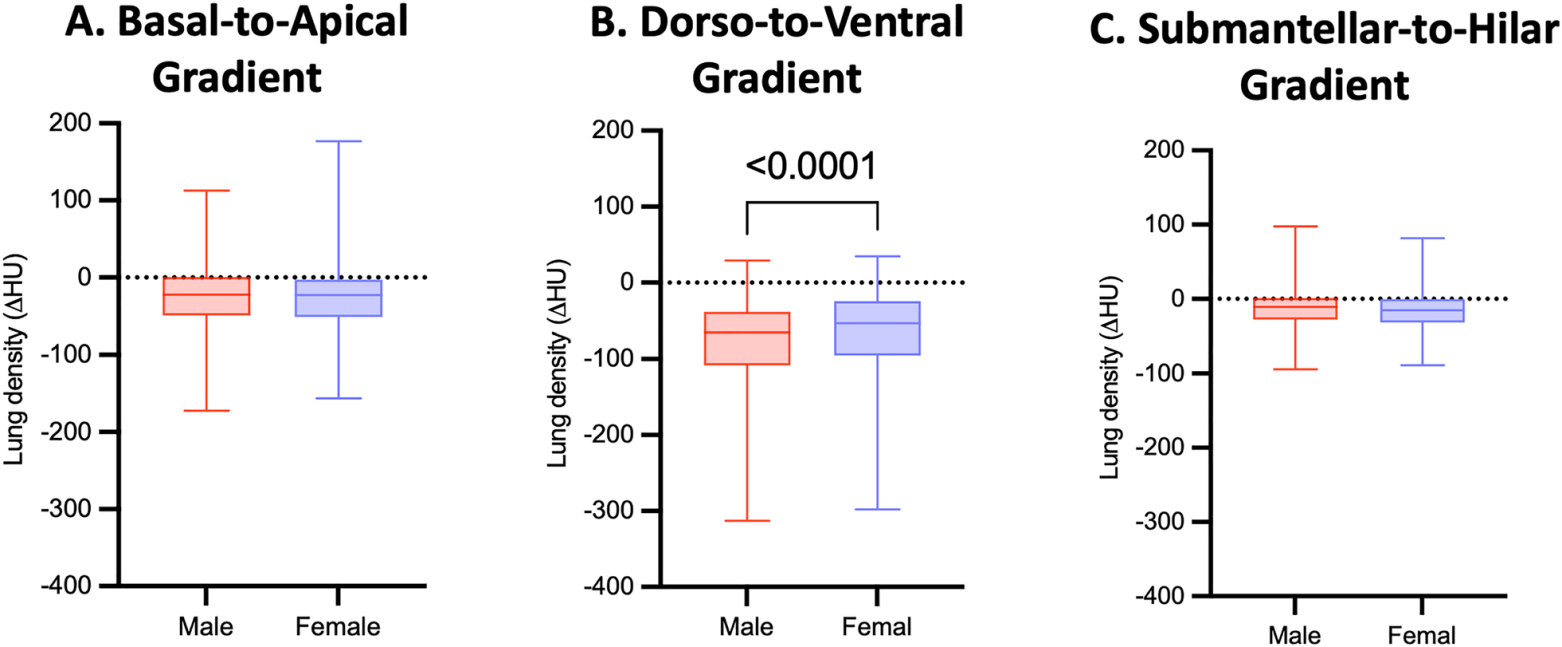
Lung density across the Basal-to-Apical (**A**), Dorso-to-Ventral (**B**), and Submantellar-to-Hilar (**C**) Gradients in male and female patients. *N* male = 554; *N* female = 296; data are presented as box and whiskers (min. to max., line at median)

Figure 3 represents the density gradients across the lung stratified by sex. The basal-to-apical density gradient was similar across sexes (−22 vs. −23 HU, *p* = 0.424). In contrast, the dorsal-to-ventral gradient was more pronounced in men (−65 vs. −53 HU, *p* < 0.001). The peripheral-to-central gradient showed a trend toward higher values in women (−15 vs. −11 HU, *p* = 0.079).

**Fig. 4:**
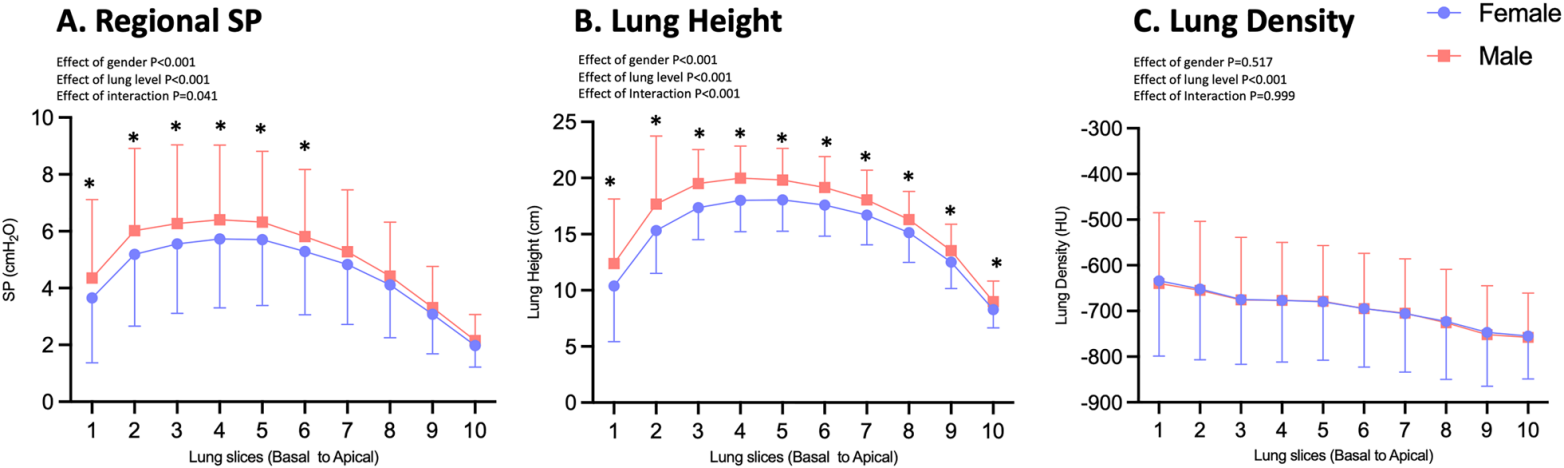
Basal-to-Apical Distribution of Regional Superimposed Pressure (**A**), Lung Height (**B**), and Lung Density (**C**) in male and female patients. *N* male = 554; *N* female = 296; data are presented as means + SD; * for *P* < 0.05 for comparison between Female and Male. Basal-to-apical regional SP was calculated by dividing the lung into 10 equally spaced, craniocaudally distributed sections (each representing one-tenth of the maximum apico-basal height); for each section, SP was obtained as the product of the CT density and the corresponding ventro-dorsal height [36] SP: Superimposed Pressure

Figure 4 shows the distribution of regional SP, lung height, and lung density across ten basal-to-apical segments in male and female subjects. At more basal levels (e.g., from first to sixth level), men exhibited higher SP values than women, and the two-way ANOVA indicated significant effects of sex (*p* < 0.001), lung level (*p* < 0.001), and their interaction (*p* = 0.041) (Fig. 4A). The ventro-dorsal lung height (Fig. 4B) was significantly greater in men compared to women across all apical–basal levels, whereas no differences between sexes were observed in lung density (Fig. 4C).

**Fig. 5:**
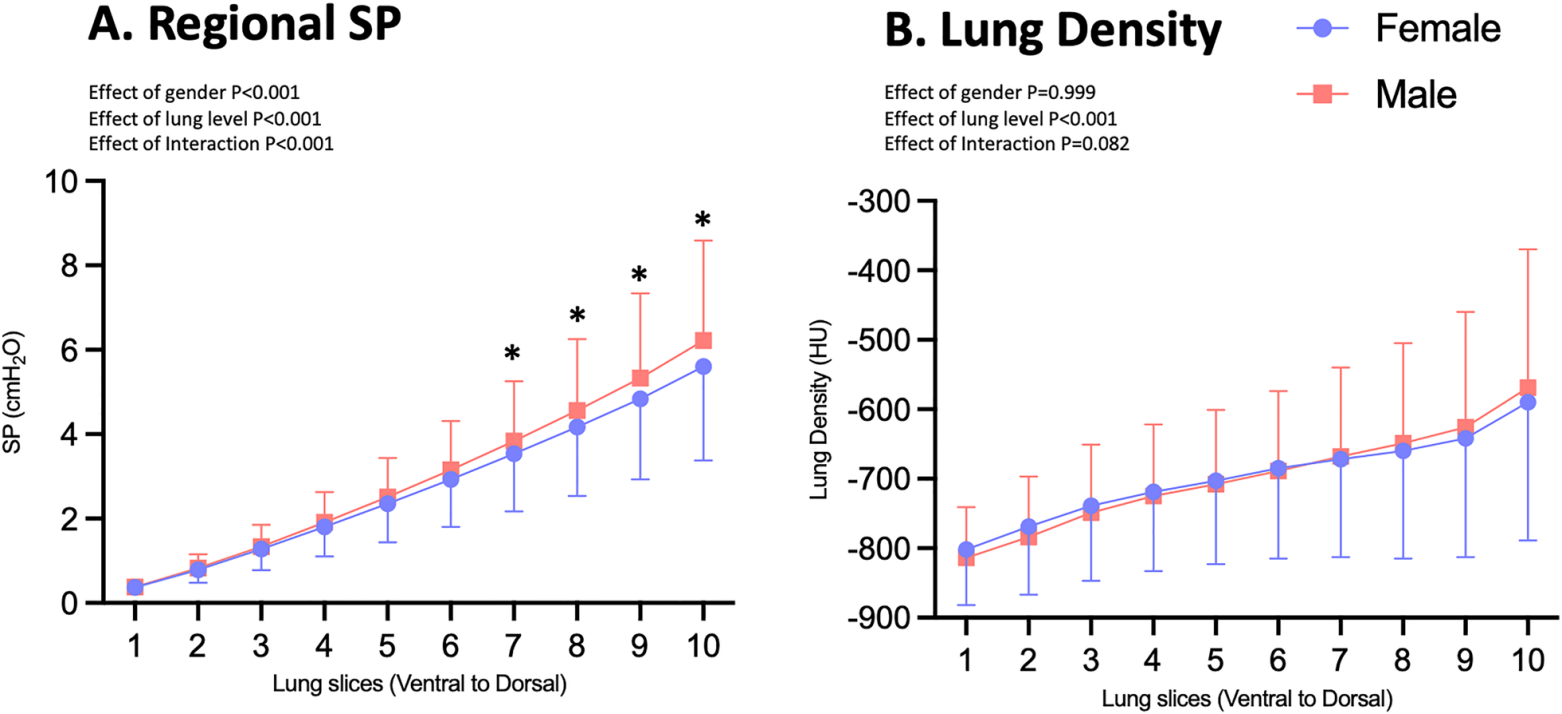
Ventral-to-Dorsal Distribution of Regional Superimposed Pressure (**A**), and Lung Height (**B**) in male and female patients. *N* male = 554; *N* female = 296; data are presented as means + SD; * for *P* < 0.05 for comparison between Female and Male. Ventral-to-dorsal regional SP was calculated by dividing the lung into 10 equally spaced, gravitationally distributed sections (each representing one-tenth of the maximum dorso-ventral lung height); for each section, SP was obtained as the product of the CT density and the section’s dorso-ventral height, cumulatively adding the SP of the sections above [1] SP: Superimposed Pressure

Figure 5 shows the distribution of regional SP and lung density across ten ventral-to-dorsal segments in male and female subjects. SP progressively increased from ventral to dorsal regions and was significantly higher in men in the seventh-to-tenth lung levels (Fig. 5A). No differences between sexes were observed in the ventral-to-dorsal distribution of lung density (Fig. 5B). When the lung was divided into dorsal, dorso-vental, and ventral compartments, men showed greater lung volumes and masses in all the compartments (all *p* < 0.001). In the dorsal region, men also had a slightly higher GGO fraction (0.44 vs. 0.41, *p* = 0.034). In ventral areas, the consolidation fraction showed a marginal but significant difference (median 0.02 in both sexes, *p* = 0.024) (Table S2).

When considering the peripheral, peripheral-centrohilar, and centrohilar lung compartments, men had greater volumes and masses (all *p* < 0.001), whereas injury, GGO, and consolidation fractions did not differ between sexes (Table S2). The extent and laterality of disease involvement also did not differ by sex. In approximately 43% of patients in both groups, COVID-19 pneumonia involved all four lung quadrants, while about one quarter patients in each group showed no radiological involvement (Table S2).

### Relationship between lung quantitative CT parameters and blood gas analysis

**Fig. 6:**
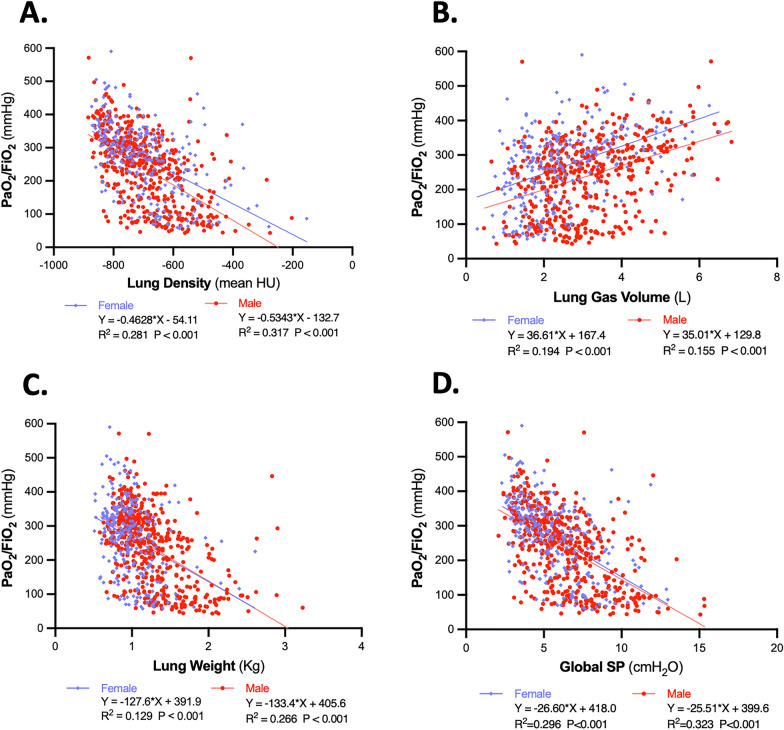
Linear correlation between Lung Density (**A**), Lung Gas Volume (**B**), Lung Weight (**C**), Global Superimposed Pressure (**D**) and oxygenation (PaO_2_/FiO_2_) in male and female patients. *N* male = 554; *N* female = 296; PaO_2_/FiO_2_: ratio between the arterial partial pressure of oxygen and the fraction of inspired oxygen; SP: Superimposed Pressure

Figure 6 shows the correlation between CT-derived parameters (lung density, lung gas volume, lung weight, and SP) and PaO_2_/FiO_2_, stratified by sex. Lung gas volume was mildly positively correlated with PaO_2_/FiO_2_, with a trend toward a stronger correlation in women. Lung density, lung weight and SP were inversely associated with oxygenation. Variations in density, lung mass, and SP explained a substantial proportion of the variability in PaO_2_/FiO_2_ (R^2^ ranging from low to moderate values, all *p* < 0.001) and the strength of these correlations showed a trend to be weaker in women.

**Fig. 7:**
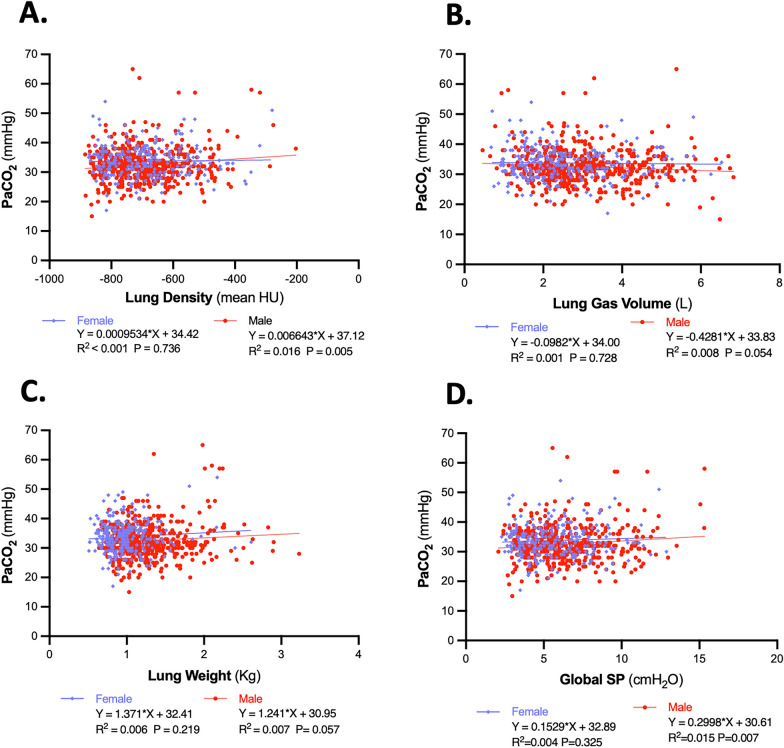
Linear correlation between Lung Density (**A**), Lung Gas Volume (**B**), Lung Weight (**C**), Global Superimposed Pressure (**D**) and PaCO_2_ in male and female patients. *N* male = 554; *N* female = 296; PaCO_2_: arterial partial pressure of carbon dioxide; SP: Superimposed Pressure

Figure 7 shows the correlation between the same CT-derived parameters and PaCO_2_. Overall, the associations between PaCO_2_ and the morphometric variables were very weak, with R^2^ values close to zero. Only in men some correlations reached or approached statistical significance, whereas in women no meaningful associations were observed.

### Outcomes and unadjusted survival analysis

**Fig. 8:**
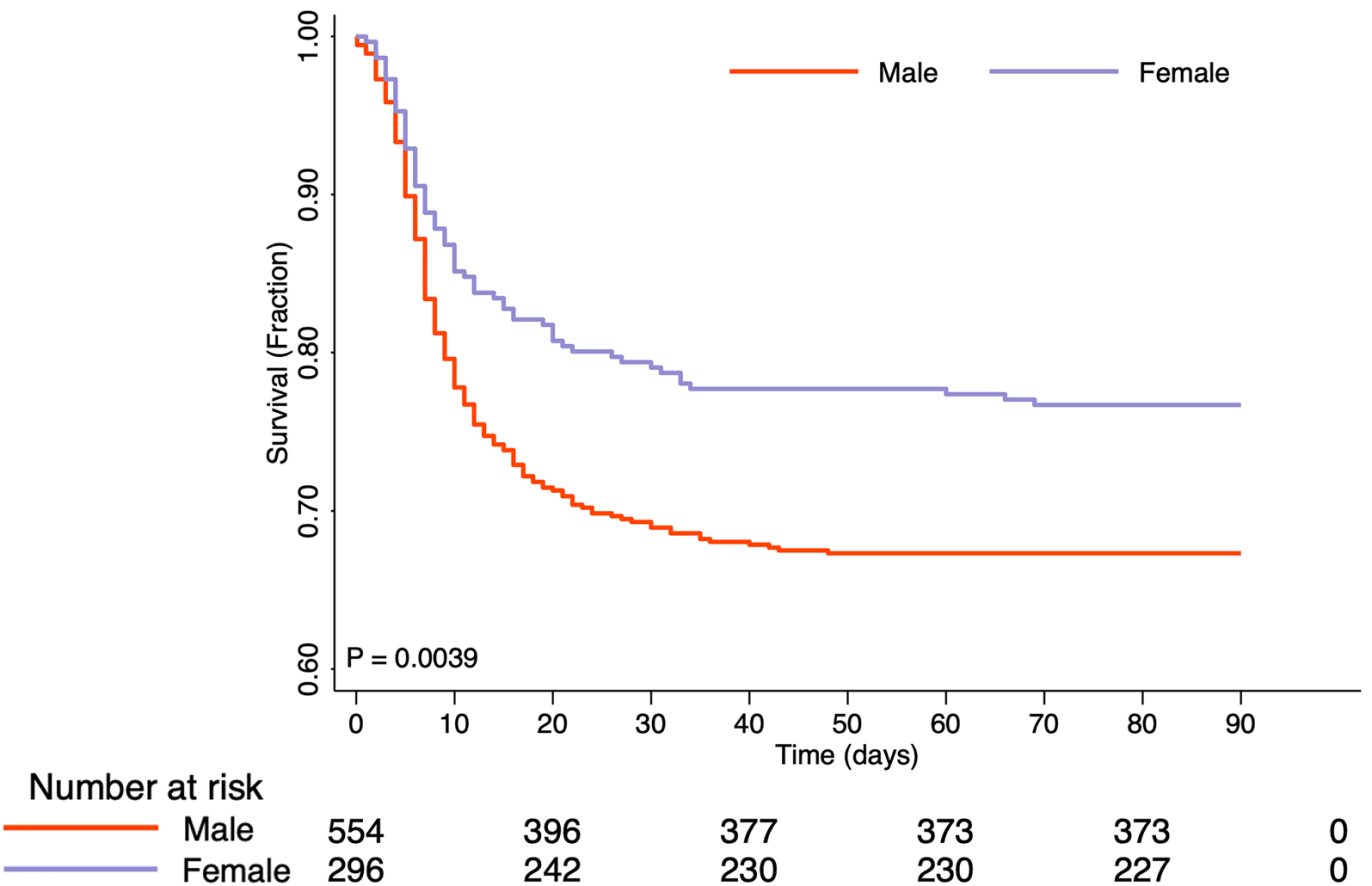
Unadjusted Kaplan–Meier survival curves in male and female patients. *N* male = 554; *N* female = 296; Differences between the two groups were assessed using log-rank test

ICU admission occurred more frequently in men than women (19% vs. 11%, *p* = 0.005). Among patients admitted to the ICU, the need for invasive respiratory support during the ICU stay was higher in men (21% vs. 14%, *p* = 0.044).Decisions to limit life-sustaining treatments were also more frequent in men (25% vs. 18%, *p* = 0.049). Both overall in-hospital mortality, and ICU mortality were significantly higher in men than in women (33% vs. 23%, *p* = 0.002 and 45% vs. 23%, *p* = 0.030, respectively). A statistically significant difference in 90-day survival between men and women was observed (*p* = 0.0039 by Log-rank test; Fig. 8).

### Multivariable analysis and mediation analysis

In our population, older age (OR 1.08, 95% CI 1.06–1.11; *p* < 0.001), higher SOFA score (OR 2.67, 95% CI 1.43-4.98; p=0.002 for SOFA ≥ 2), lower PaO_2_/FiO_2_ (OR 0.99, 95% CI 0.98–0.99; *p* < 0.001), higher global SP (OR 1.18, 95% CI 1.05–1.34; *p* = 0.005), and male sex (OR 1.76, 95% CI 1.06–2.92; *p* = 0.028) were independently associated with an increased risk of overall in-hospital mortality (Table 1). We also performed a mediation analysis (Table 2) to evaluate whether the independent association between male sex and mortality identified in the multivariable analysis, was mediated by the higher global SP observed in men. The results indicate that global SP is a strong predictor of mortality in our population (coefficient 0.31, 95% CI 0.23–0.37; *p* < 0.001), with a trend toward a significant association between sex and outcome (coefficient 0.33, 95% CI −0.01–0.67; *p* = 0.057). However, the effect of global SP on mortality does not appear to be mediated by male sex (coefficient 0.00).

**Table 1:** Multivariable analysis of factors independently associated with probability of in-hospital mortality in the overall population

Variable	OR	95% CI	*p* value
Age (1 year increase)	1.08	1.06–1.11	< 0.001
Any comorbidities (ref. No)	1.65	0.93–2.93	0.085
Composite SOFA score			
1 (ref. 0)	2.32	1.37–3.93	0.002
≥ 2 (ref. 0)	2.67	1.43–4.98	0.002
PaO_2_/FiO2 (1 mmHg)	0.99	0.98–0.99	< 0.001
White blood cell (1*10^3^)	0.97	0.92–1.03	0.349
Positive pressure ventilation (ref. No)	2.52	0.68–9.36	0.168
Global superimposed pressure (1 cmH_2_O)	1.18	1.05–1.34	0.005
Sex (ref. female sex)	1.76	1.06–2.92	0.028

Positive pressure ventilation refers to non-invasive respiratory support (e.g., CPAP or BiPAP) applied prior to CT acquisition. *N* = 614. PaO_2_/FiO_2_: ratio of arterial oxygen partial pressure to fraction of inspired oxygen; SOFA: sequential organ failure assessment

**Table 2:** Mediation analysis of the association between global superimposed pressure and mortality, considering sex as a potential mediator

	Coefficient	Std. err	95% CI	*p* value
Mortality				
• Global superimposed pressure	0.33	0.17	−0.01–0.67	0.057
• Sex (ref. female sex)	0.31	0.03	0.23–0.37	< 0.001
Global superimposed pressure				
• Sex (ref. female sex)	0.00	/	/	/

*N* = 850

In addition, when testing multivariable models, including either global SP or, alternatively, male sex, both variables remained independently associated with mortality (Tables S4–S6) However, formal testing of the interaction between global superimposed pressure and sex did not show a statistically significant effect (OR 1.20, 95% CI 0.94–1.53; *p* = 0.135) (Table S7).

Finally, we tested the potential role of lung height as a confounding factor linking sex to lung dimensions and global SP. Additional multivariable analyses including height as covariate (Tables S8, S9) showed that global SP and male sex remained significantly associated with mortality, whereas height was not independently associated with the outcome (OR 1.01, 95% CI 0.91–1.21; *p* = 0.796, and OR 0.95, 95% CI 0.84–1.06; *p* = 0.369 in the models, including global SP, and both global SP and male sex, respectively).

## Discussion

In this large multicenter cohort of patients with COVID-19-related AHRF, we identified significant sex-related differences in lung morphology, mechanical load, and outcomes derived from quantitative CT analysis. Our main findings can be summarized as follows:

- Men exhibited larger lung volumes, greater lung mass, and higher global SP compared with women, despite similar degrees of radiological injury quantified as ground-glass opacities or consolidation.
- Higher global SP and male sex, independently predicted a higher mortality rate alongside age, oxygenation, and SOFA score.
- The effect of global superimposed pressure on mortality does not appear to be mediated by sex.

These findings indicate that SP, a CT-derived estimate of gravitational stress and compressive forces within the lung parenchyma [10, 37], captures pathophysiological differences between men and women with COVID-19 [23, 38].

Our results extend prior work from the same CT-COVID19 multicenter dataset, demonstrating that SP not only reflects the severity of lung injury and oxygenation impairment but also carries significative outcome prediction.

Our data suggest that male patients may experience greater gravitational loading and mechanical disadvantage during spontaneous breathing, potentially contributing to more severe gas exchange abnormalities and poorer clinical outcomes.

Several mechanisms may explain the observed sex differences in CT-derived lung morphology, and their potential impact on disease severity and outcomes. We found that men had greater lung height compared with women. This configuration may lead to a greater hydrostatic gradient and, consequently, to higher global SP values for the same degree of lung density. When dividing the lung into ten ventral-to-dorsal levels, regional SP differed between men and women in the dorsal lung region. Given that lung height at the level of each segment (maximum anteroposterior diameter divided by ten) was constant across the ventro-dorsal axis, and that lung density did not differ significantly between sexes, other factors—such as the mechanical load applied by the chest-wall or its geometric configuration [35]—may contribute the higher SP observed in men.

This structural configuration may predispose the male lung to increased compressive stress and gravitational collapse of basal and dorsal regions, amplifying regional inhomogeneity and mechanical load even without overt differences in total radiological injury. In contrast, women—with shorter lung height and lower lung mass—may experience a more homogeneous distribution of stress and strain along both the apical–basal and ventro-dorsal axes, possibly mitigating the mechanical disadvantage imposed by gravity.

We found that women with COVID-19-related AHRF had a higher probability of 90-day survival compared with men. In addition, male sex and global SP—together with older age, higher SOFA score, and lower PaO_2_/FiO_2_ ratio—were independently associated with mortality. The independent association between higher risk of mortality and global SP, has a strong physiological rationale, as SP reflects the weight of the edematous lung applying on dependent regions, and contributing to derecruitment and hypoxemia [1, 38].

Global SP integrates both morphological and mechanical information due to gravitational stress and tissue load, that may have a greater pathophysiological impact in the male lung, partially explaining the higher mortality observed in men. In women, despite similar degrees of radiological injury, lower global SP values may indicate a smaller mechanical burden for comparable parenchymal involvement, consistent with the lower mortality observed across multiple COVID-19 and ARDS cohorts [20, 22]. These findings, consistent with recent literature, emphasize the importance of accounting for body size in both ventilation settings [39] and outcome stratification [40].

The mediation analysis supports the role of global SP as a strong predictor of mortality in our population. However, the effect of global SP on mortality does not appear to be mediated by male sex. In addition, the interaction between global SP and sex did not show a significant effect. Together these results suggest that the association between global SP and mortality does not differ significantly between males and females and that global SP should be interpreted as an independent predictor of mortality rather than a sex-specific prognostic factor. Therefore, factors beyond anatomical characteristics associated with male sex (e.g., greater lung height), may exert a direct effect on global SP. The absence of a significative association between lung height and outcome further supports this finding.

The significative association between increased risk of mortality and male sex, may be mediated by biological (e.g., systemic inflammation) or hormonal factors. Estrogen and progesterone have been shown to exert protective effects on vascular permeability and inflammation through modulation of endothelial function and cytokine release [26]. Experimental and clinical data indicated that female sex is associated with enhanced innate and adaptive immune responses, reduced pulmonary inflammation, and greater alveolar epithelial repair capacity [24]. These mechanisms may limit pulmonary edema and tissue density increase, indirectly attenuating SP. Conversely, testosterone has been associated with increased vascular permeability and inflammatory responses, promoting lung fluid overload and amplifying the development of dependent atelectasis.

Our results extend and integrate previous evidence on physiological and prognostic role of CT-derived indices in acute respiratory failure. In a prior analysis of the same CT-COVID19 multicenter cohort, CT-derived variables, including mean lung density as excellent variables for subphenotyping non-intubated patients providing population enrichment and a robust independent association with mortality [15]. The current study builds upon those results by showing that SP also carries a sex-specific prognostic significance, identifying male patients as particularly vulnerable to the adverse effects of increased gravitational stress.

Sex differences in outcomes have long been recognized in ARDS and COVID-19. In a secondary analysis [41] of the LUNG SAFE study [21], although the overall hospital mortality was similar between sexes, female sex was associated with higher mortality in patients with severe ARDS, likely due to less exposure to protective ventilation. During the COVID-19 pandemic, several large-scale studies confirmed that men faced higher risks of hospitalization, respiratory failure, and death compared with women [22, 23]. However, most of these investigations focused on epidemiological or immunological factors. This study adds imaging-based evidence suggesting that differences in lung morphology and mechanical load distribution, quantified through global SP, may only partially explain these outcome disparities.

Furthermore, our findings complement recent imaging studies employing artificial intelligence and clustering analyses to identify CT-based sub-phenotypes in COVID-19 acute respiratory failure [15, 17, 42]. By incorporating biological variables, such as sex into the interpretation of CT-derived mechanical metrics, we highlight how morphological quantification can refine patient phenotyping implementing precision medicine approaches.

As quantitative CT analysis becomes increasingly automated through deep-learning algorithms, SP estimation can be rapidly obtained from routine chest CT scans. This opens the possibility for real-time risk stratification and individualized management strategies, potentially guiding ventilation settings or positional therapy based on both sex and mechanical load distribution. The recognition of SP as a modifiable correlate of outcome may, therefore, bridge imaging, physiology, and personalized critical care in acute respiratory failure.

These findings may have relevant clinical implications for the assessment and management of AHRF. SP, as a quantitative CT-derived marker, integrates the effects of lung density, geometry, and gravitational gradients into a single parameter. Its association with outcome supports the concept that greater gravitational load and inhomogeneity may predispose to regional collapse and self-inflicted lung injury during spontaneous breathing.

From a translational standpoint, SP quantification could serve as an imaging biomarker of mechanical vulnerability, complementing traditional physiological parameters, such as PaO₂/FiO₂ or lung compliance, and helping to identify individuals at increased risk of disease progression or need for ventilatory support[43].

This study has several limitations. First, the retrospective design of the study implies missing data and potential unmeasured confounders. Although imaging and clinical data were collected prospectively across multiple centers, differences in CT acquisition protocols and patient positioning may have introduced variability in SP estimation. Second, the study population was enrolled during the first wave of the COVID-19 pandemic [44], when therapeutic approaches were heterogeneous and vaccination was unavailable [45]; thus, our findings may not fully generalize to more recent cohorts or to non-COVID-19 causes of AHRF [46]. Third, the CT assessment did not allow evaluation of dynamic SP changes or its modulation by clinical interventions, such as non-invasive ventilation or prone positioning. Fourth, the ten vertical layers approach to calculate SP assumes fluid-like lung behavior and may overlook local stress concentrations or confounding factors, such as increased intra-abdominal pressure. Finally, we did not have direct measurements of inspiratory effort (e.g., esophageal pressure), limiting assessment and contribution of self-inflicted lung injury.

Despite these limitations, the strengths of our work include its large, multicenter design, the rigorous manual validation of automated CT segmentations, and the homogeneous inclusion of spontaneously breathing patients early in their disease course.

Future studies should aim to prospectively validate these results and assess whether SP-guided management strategies—such as early pronation or targeted ventilatory support—may improve clinical outcome. Integrating imaging-derived mechanical metrics with omics and immune profiling could further elucidate the biological pathways linking sex, lung mechanics, and outcome in AHRF.

## Conclusions

In summary, our findings suggest that biologic sex may modulate the relationship between lung morphology, mechanics, and outcomes in COVID-19-related AHRF. Male patients exhibited larger lung size and higher SP values, reflecting a higher hydrostatic load imposed by the weight of the lung along the gravitational axis. Both global SP and male sex were independently associated with mortality, underscoring that beyond anatomical and mechanical differences, biological and hormonal factors likely contribute to the increased disease severity observed in men. Overall, SP appears to be a promising CT-derived biomarker of lung injury severity and mechanical vulnerability. By integrating quantitative imaging with physiological assessment, SP may help refine personalized ventilatory strategies and advance precision medicine approaches in the management of AHRF.

## Supplementary Information

Supplementary Material 1

## Data Availability

The data that support the findings of this study are not publicly available, because they contain information that could compromise the privacy of research participants. However, they are available from the corresponding authors upon request.

## Abbreviations

AHRF: Acute hypoxemic respiratory failure
AIC: Akaike information criterion
ANOVA: Analysis of variance
ARDS: Acute respiratory distress syndrome
ARF: Acute respiratory failure
AUROC: Area under the receiving operating characteristics
BIC: Bayesian information criterion
BMI: Body mass index
CI: Confidence interval
COPD: Chronic obstructive pulmonary disease
CNN: Convolutional neural network
CT: Computed tomography
FiO_2_: Inspiratory oxygen fraction
GGO: Ground glass opacity
HU: Hounsfield Unit
ICU: Intensive care unit
IQR: Interquartile range
LA: Left apical
LB: Left basal
LOS: Length of stay
PaCO_2_: Arterial carbon dioxide partial pressure
PaO_2_: Arterial oxygen partial pressure
pH: Negative logarithm of hydrogen concentration
RA: Right apical
RB: Right basal
ROI: Region of interest
SD: Standard deviation
SOFA: Sequential organ failure assessment
