# Supplementary material for: Sex-related differences in lung injury distribution and outcomes in COVID-19 acute respiratory failure: insights from the CT-COVID19 multicenter study group
Source: Intensive Care Med Exp. 2026 Jun 16;14:76. doi: 10.1186/s40635-026-00914-4 (PMC13272716; doi:10.1186/s40635-026-00914-4)
Supplement: Supplementary file 1 [file 40635_2026_914_MOESM1_ESM.docx]

SUPPLEMENTARY MATERIAL

Sex-Related Differences in Lung Injury distribution and Outcomes in COVID-19 Acute Respiratory Failure: Insights from the CT-COVID19 Multicenter Study Group

Running Head: Sex and Lung CT in COVID-19 Respiratory Failure

Authors

Davide Signori1*, Alice Nova2,3*, Yi Xin4,5, Sarah E. Gerard6, Aurora Magliocca7,8, Giovanni Vitale7, Linda Mussoni9, Jonathan Montomoli10,11, Matteo Subert12, Alessandra Ponti7, Savino Spadaro13,14, Giancarla Poli15, Francesco Casola16,17, Roberta Garberi2,3, Andrea Restivo2,3, Davide Raimondi Cominesi2,3, Marco Giani2,3, Vanessa Zambelli2, Giuseppe Foti2,3, John G. Laffey18,19, Maurizio Cereda#4,5, Emanuele Rezoagli#2,3, and for the CT-COVID19 Multicenter Study Group

CT-COVID19 multicenter study group collaborators:

Department of Anaesthesia and Critical Care Medicine, Papa Giovanni XXIII Hospital, Bergamo, Italy: Pietro Bonaffini, Matteo Cazzaniga, Ferdinando Luca Lorini;

Department of Translational Medicine, University of Ferrara, Ferrara, Italy: Irene Ottaviani;

Department of Anesthesiology and Intensive Care, ASST Lecco, Lecco, Italy: Asia Borgo, Mario Tavola;

Department of Anesthesia and Intensive Care Medicine, Melzo-Gorgonzola Hospital, Azienda Socio-Sanitaria Territoriale Melegnano e della Martesana, Melegnano, Milan, Italy: Livio Ferraris;

Department of Emergency and Intensive Care and Department of Radiology, Fondazione IRCCS San Gerardo dei Tintori Hospital, Monza, Italy: Giacomo Bellani, Stefano Gatti, Davide Ippolito, Filippo Serra;

Istituto per la Sicurezza Sociale, San Marino, San Marino: Massimo Arlotti, Marino Gatti, Beatrice Tamagnini;

Department of Anesthesia and Intensive Care, Infermi Hospital, AUSL Romagna, Rimini, Italy: Enrico Cavagna, Emiliano Gamberini;

Department of Anesthesia and Intensive Care Medicine, Policlinico San Marco, Gruppo Ospedaliero San Donato, Bergamo, Italy: Davide De Ponti, Giuseppe Galbiati, Matteo Giacomini.

*Davide Signori and Alice Nova shared co-first authorship

#Emanuele Rezoagli and Maurizio Cereda shared co-senior authorship

Author Affiliations

1 Department of Anesthesia and Intensive Care, Azienda Socio Sanitaria Territoriale Bergamo Est, Seriate, Italy

2 School of Medicine and Surgery, University of Milano-Bicocca, Monza, Italy

3 Department of Emergency and Intensive Care, Fondazione IRCCS San Gerardo dei Tintori Hospital, Monza, Italy

4 Department of Anesthesiology, Critical Care, and Pain Medicine, Massachusetts General Hospital, Harvard Medical School, Boston, Massachusetts, USA

5 Department of Anesthesiology and Critical Care, University of Pennsylvania, Philadelphia, USA

6 Roy J. Carver Department of Biomedical Engineering, University of Iowa, Iowa City, Iowa, USA

7 Department of Anesthesia and Intensive Care Medicine, Policlinico San Marco, Gruppo Ospedaliero San Donato, Bergamo, Italy

8 Department of Pathophysiology and Transplantation, University of Milan, Milan, Italy

9 Istituto per la Sicurezza Sociale, San Marino, San Marino, Italy

10 Department of Anesthesia and Intensive Care, Infermi Hospital, AUSL Romagna, Rimini, Italy

11 Health Services Research, Evaluation and Policy Unit, Romagna Local Health Authority, Rimini, Italy

12 Department of Anesthesia and Intensive Care Medicine, Melzo-Gorgonzola Hospital, Azienda Socio-Sanitaria Territoriale Melegnano e della Martesana, Melegnano, Milan, Italy

13 Department of Anesthesia and Intensive Care Medicine, Azienda Ospedaliero-Universitaria of Ferrara, Ferrara, Italy

14Department of Translational Medicine, University of Ferrara, Ferrara, Italy

15 Department of Anaesthesia and Critical Care Medicine, Papa Giovanni XXIII Hospital, Bergamo, Italy

16 Department of Physics, Harvard University, Cambridge, Massachusetts, USA

17 Harvard-Smithsonian Centre for Astrophysics, Cambridge, Massachusetts, USA

18 School of Medicine, National University of Ireland Galway, Galway, Ireland

19 Department of Anaesthesia and Intensive Care Medicine, Galway University Hospitals, Galway, Ireland

### Materials and Methods

Ethical consideration and data acquisition 

The study was performed under the Declaration of Helsinki and in agreement with the Italian good clinical practice recommendations (D.M. Sanità del 15/07/97 e s.m.i.) and with the applied healthcare hospital protocols, as we previously described (1). No change of current clinical practice or clinical protocols in use were taken in place in the enrolled study population. Considering the retrospective nature of the proposed study, we did not anticipate risks nor benefits that might be added to the patients. Moreover, in the presence of technical difficulties related to the emergency health context to obtain an informed consent from patients in that period of pandemic, informed con- sent was waived. For this reason and for the great public interest of the project, the research was conducted in the context of the authorizations guaranteed by Article 89 of the GDPR EU Regulation 2016/679, which guaran- tees the treatment for purposes of public interest, scientific or historical research or for statistical purposes of health data. Personal data were handled in compliance with the European Regulation on the Protection of Personal Data (GDPR), the Legislative Decree 196/2003 and subsequent amendments and additions, and any other Italian law applicable to the protection of personal data (henceforth referred to as the “applicable data protection law”). Data were collected in a pseudo-anonymous way through paper case report forms, digitalized on a University of Milano-Bicocca Institutional Google drive account and analyzed by the scientific coordinator of the project (E.R.). Favorable judgment for the execution of the study was obtained before data acquisition from the local institutional review board of the coordinating center Fondazione IRCCS San Gerardo dei Tintori, Monza, Italy (Approval date: 24/04/2020; number 3375) and from the local institutional review board of each enrolled center (Policlinico San Marco, Gruppo Ospedaliero San Donato, Zingonia, Bergamo, Italy; Ospedale Infermi, Rimini, Italy; Ospedale Papa Giovanni XXIII, Bergamo, Italy; Ospedale Alessandro Manzoni, Lecco, Italy; Arcispedale Sant’Anna, Ferrara, Italy; Ospedale Santa Maria delle Stelle, Melzo, Italy; Istituto Sicureza Sociale, Repubblica di San Marino). Baseline characteristics (age, sex, body mass index, comorbidities) and clinical illness severity (Sequential Organ Failure Assessment (SOFA) and pH) were collected, together with laboratory biomarkers, blood gas analysis, respiratory assistance, and hemodynamic data at hospital admission. Lung CT scans acquired for clinical purposes within the first week since hospital admission were obtained. Data on drug treatments and complications during hospital admissions, outcomes including length of stay (in ICU and in hospital), use of non-invasive respiratory support, mechanical ventilation-free days, limitation of life sustaining measures, ICU mortality, and hospital mortality were recorded.                               

| Variable | N | OR | 95% CI | p |
| --- | --- | --- | --- | --- |
| Age (1 year increase) | 850 | 1.08 | 1.06-1.09 | <0.001 |
| Any comorbidities (Ref. No) | 850 | 3.28 | 2.29-4.71 | <0.001 |
| Creatinine (1 mg/dL) | 826 | 2.20 | 1.72-2.83 | <0.001 |
| Bilirubin (1 mg/dL) | 686 | 1.48 | 0.96-2.29 | 0.076 |
| Platelets (n*103) | 829 | 0.99 | 0.99-1.00 | 0.588 |
| PaO2/FiO2 (1 mmHg) | 744 | 0.99 | 0.98-0.99 | <0.001 |
| White Blood Cells (n*103) | 831 | 1.08 | 1.04-1.12 | <0.001 |
| CRP (1 mg/dL) | 811 | 1.06 | 1.04-1.08 | <0.001 |
| Limitation of care (Ref. No) | 731 | 232.35 | 92.08-586.33 | <0.001 |
| Positive Pressure Ventilation (Ref. No) | 827 | 3.85 | 1.77-8.34 | 0.001 |
| Global Superimposed Pressure (1 cmH2O) | 850 | 1.37 | 1.28-1.46 | <0.001 |
| Sex (Ref. Female) | 850 | 1.62 | 1.18-2.24 | 0.003 |
| Composite SOFA score 1 (ref. 0)               ≥ 2 (ref. 0) | 683 | 3.24                4.61 | 2.20-4.77   2.90-7.34 | <0.001 <0.001 |

Table S1. Exploratory univariable analysis of factors which may be independently associated with probability of mortality in the overall population. Positive pressure ventilation refers to non-invasive respiratory support (e.g., CPAP or BiPAP) applied prior to CT acquisition. List of abbreviation: CRP: C-reactive protein; PaO₂ /FiO₂: ratio of arterial oxygen partial pressure to fraction of inspired oxygen; SOFA: Sequential Organ Failure Assessment.

Results

Tables

|  | Overall N = 850 | Female N=296 (35%) | Male N=554 (65%) | P-value |
| --- | --- | --- | --- | --- |
| Reason of hospital admission |  |  |  |  |
| Respiratory failure, n (%) | 850 (100) | 296 (100) | 554 (100) | - |
| Baseline characteristics |  |  |  |  |
| BMI, kg/m2; N=359 | 27.5 [24.7;0.5] | 27.3 [24.3;31.2] | 27.6 [24.7;30.1] | 0.635 |
| Time between admission and CT scan, days | 0 [0;0] | 0 [0;0] | 0 [0;1] | 0.138 |
| Comorbidities |  |  |  |  |
| COPD, n (%) | 50 (6) | 15 (5) | 35 (6) | 0.461 |
| Asthma, n (%) | 34 (4) | 16 (5) | 18 (5) | 0.126 |
| Congestive heart failure, n (%) | 36 (4) | 9 (3) | 27 (5) | 0.206 |
| Chronic kidney disease, n (%) | 51 (6) | 12 (4) | 39 (7) | 0.081 |
| Chronic liver failure, n (%) | 5 (0.6) | 2 (0.7) | 3 (0.5) | 0.807 |
| Immune mediated disease, n (%) | 32 (4) | 18 (6) | 14 (2) | 0.010 |
| Diabetes, n (%) | 143 (17) | 44 (15) | 99 (18) | 0.264 |
| Systemic hypertension, n (%) | 419 (49) | 145 (49) | 274 (49) | 0.896 |
| OSAS, n (%) | 13 (1.5) | 0 (0) | 13 (2) | 0.008 |
| Thyroid dysfunction, n (%) | 36 (6) | 22 (11) | 14 (3.5) | <0.001 |
| Any comorbidities, n (%) | 556 (65) | 190 (64) | 366 (66) | 0.584 |
| Clinical illness severity |  |  |  |  |
| pH, N=732 | 7.47 [7.44;7.49] | 7.47 [7.43;7.49] | 7.47 [7.44;7.49] | 0.761 |
| PaO2/FiO2, mmHg, N=744 | 263 [160;323.5] | 282 [187;340] | 255 [145;315] | <0.001 |
| PaCO2 (mmHg); N=742 | 32 [29;36] | 33 [30;37] | 32 [29;36] | <0.001 |
| Bicarbonate, mEq/L, N=731 | 22.8 [20.9;24.7] | 23.4 [21.4;25.3] | 22.7 [20.6;24.5] | 0.003 |
| D-Dimer, µg/mL, n=376 | 0.95 [0.57;1.99] | 0.94 [0.66;2.06] | 0.95 [0.56;1.87] | 0.623 |
| Respiratory support, N=827 | Room Air, n (%) | 527 (64) | 196 (69) | 331 (61) | 0.095 |
| Low Flow Oxygen, n (%) | 272 (33) | 79 (28) | 193 (35) |
| NIMV – BiPAP, n (%) | 1 (0.1) | 0 (0) | 1 (0.2) |
| NIMV – CPAP, n (%) | 23 (3) | 8 (3) | 15 (3) |
| IMV, n (%) | 4 (0.5) | 0 (0) | 4 (0.7) |
| Laboratory parameters at hospital admission |  |  |  |  |
| WBCs (x1000/μL); N=831 | 6.5 [4.9;9.0] | 6.2 [4.8;8.3] | 6.7 [4.9;9.3] | 0.021 |
| Platelets (x1000/μL); N=829 | 188 [147;248] | 204 [163;281] | 180 [139;238] | <0.001 |
| INR; N=753 | 1.04 [0.99;1.14] | 1.02 [0.98;1.10] | 1.06 [1.00;1.15] | <0.001 |
| Tn-I (ng/ml); N=378 | 0.20 [0.20;0.18] | 0.03 [0.02;0.44] | 0.20 [0.20;0.13] | 0.077 |
| LDH (UI/l); N=511 | 379 [272;517] | 354 [245;515] | 392.5 [283;521] | 0.056 |
| CPK (UI/l); N=398 | 134 [72;296] | 102.5 [49;246] | 149.5 [85;308] | 0.001 |
| IL-6 (pg/ml); N=19 | 82.4 [46.8;148.0] | 46.8 [36.4;83.6] | 102.2 [54.9;149.0] | 0.130 |
| Bilirubin total (mg/dl); N=686 | 0.56 [0.40;0.76] | 0.47 [0.34;0.61] | 0.61 [0.45;0.82] | <0.001 |
| Creatinine (mg/dl); N=826 | 1.06 [0.88;1.38] | 0.87 [0.72;1.12] | 1.12 [0.97;1.46] | <0.001 |
| Temperature (°C); N=824 | 37.6 [36.8;38.3] | 37.45 [36.6;38.05] | 37.7 [36.9;38.3] | 0.002 |
| Complications |  |  |  |  |
| All Bacterial overinfections, n (%); N=830 | 121 (15) | 35 (12) | 86 (16) | 0.141 |
| Venous thromboembolism, n (%); N=830 | 17 (2) | 2 (1) | 15 (3) | 0.045 |
| Pulmonary thromboembolism, n (%); N=809 | 27 (3) | 6 (2) | 21 (4) | 0.157 |
| Pneumothorax, n (%); N=850 | 10 (1) | 1 (0.3) | 9 (2) | 0.097 |
| Outcomes |  |  |  |  |
| Limitation of care, n (%); N=730 | 163 (22) | 47 (18) | 116 (25) | 0.049 |
| ICU admission, n (%); N=850 | 139 (16) | 34 (11) | 105 (19) | 0.005 |
| Endotracheal intubation during ICU stay, n (%); N=647 | 119 (18) | 30 (14) | 89 (21) | 0.044 |
| ICU mortality, n (%); N=131 | 53 (40) | 7 (23) | 46 (45) | 0.030 |
| Hospital mortality, n (%); N=811 | 190 (23) | 57 (20) | 133 (25) | 0.106 |
| Overall mortality, n (%), N=850 | 252 (30) | 69 (23) | 183 (33) | 0.002 |

Table S2. Baseline characteristics, clinical illness severity, and respiratory support at hospital admission stratified by sex. Continuous data are presented as mean ± SD or median [IQR], as appropriate; categorical variables are reported as count (proportion). P for T-test, Mann–Whitney Test or Chi square test, as appropriate. List of abbreviations: ALT: alanine aminotransferase; aPTT: activated partial thromboplastin time; AST: aspartate aminotransferase; BMI: body mass index; COPD: chronic obstructive pulmonary disease; CPAP: continuous positive airway pressure; CPK: creatine phosphokinase; CRRT: continuous renal replacement therapy; GT (GGT): gamma-glutamyl transferase; ICU: intensive care unit; IL-6: interleukin-6; INR: international normalized ratio; LDH: lactate dehydrogenase; LOS: length of stay; OSAS: obstructive sleep apnea syndrome; PaCO₂: arterial partial pressure of carbon dioxide; PaO₂ /FiO₂: ratio of arterial oxygen partial pressure to fraction of inspired oxygen; Tn-I: troponin I; Tn-T: troponin T; V-A ECMO: veno-arterial extracorporeal membrane oxygenation; V-V ECMO: veno-venous extracorporeal membrane oxygenation. 

|  | Overall N = 850 | Female N=296 (35%) | Male N=554 (65%) | P-value |
| --- | --- | --- | --- | --- |
|  |  |  |  |  |
| Whole lung |  |  |  |  |
| Lung density (HU) | 716 [-788;-632] | -715 [-798;-638] | -716 [-783;-631] | 0.691 |
| Lung gas volume (L) | 2.68 [2.01;3.73] | 2.28 [1.63;3.29] | 2.91 [2.21;3.94] | <0.001 |
| Lung mass (kg) | 1.05 [0.88;1.30] | 0.93 [0.77; 1.08] | 1.14 [0.95; 1.41] | <0.001 |
| Total injury, fraction | 0.41 [0.26;0.57] | 0.40 [0.23; 0.57] | 0.42 [0.27;0.58] | 0.412 |
| GGO, fraction | 0.35 [0.22;0.48] | 0.34 [0.20;0.47] | 0.36 [0.23; 0.48] | 0.265 |
| Consolidation, fraction | 0.05 [0.03;0.09] | 0.05 [0.03;0.09] | 0.05 [0.03;0.09] | 0.765 |
| Lung height, cm | 19.98 ± 2.13 | 19.70 ± 1.93 | 20.66 ± 1.90 | <0.001 |
| SP, cmH2O | 5.60 [4.19;7.37] | 5.21 [3.86;6.84] | 5.79 [4.35;7.67] | <0.001 |
|  |  |  |  |  |
| Basal |  |  |  |  |
| Lung density (HU) | -694 [-779;592] | -688 [-785; -691] | -695 [-775; -592] | 0.979 |
| Lung gas volume (L) | 0.86 [0.62;1.19] | 0.73 [0.51;1.06] | 0.92 [0.69;1.27] | <0.001 |
| Lung mass (kg) | 0.36 [0.30;0.46] | 0.32 [0.27;0.39] | 0.40 [0.33;0.51] | <0.001 |
| Total injury, fraction | 0.46 [0.28;0.64] | 0.46 [0.26;0.65] | 0.47 [0.29;0.64] | 0.749 |
| GGO, fraction | 0.38 [0.24;0.51] | 0.38 [0.22;0.51] | 0.39 [0.25;0.50] | 0.528 |
| Consolidation, fraction | 0.05 [0.03;0.11] | 0.05 [0.03;0.11] | 0.05 [0.03;0.10] | 0.710 |
| Basal-Apical |  |  |  |  |
| Lung density (HU) | -712 [-785;-621] | -712 [-791;-618] | -712 [-782;-622] | 0.602 |
| Lung gas volume (L) | 0.87 [0.66;1.23] | 0.75 [0.53;1.08] | 0.95 [0.73;1.30] | <0.001 |
| Lung mass (kg) | 0.36 [0.30;0.46] | 0.31 [0.26;0.37] | 0.39 [0.32;0.49] | <0.001 |
| Total injury, fraction | 0.42 [0.25,0.58] | 0.40 [0.23;0.58] | 0.43 [0.25;0.59] | 0.331 |
| GGO, fraction | 0.35 [0.21;0.47] | 0.33 [0.19;0.47] | 0.36 [0.22;0.47] | 0.176 |
| Consolidation, fraction | 0.05 [0.03;0.09] | 0.05 [0.03;0.10] | 0.05 [0.03;0.] | 0.608 |
| Apical |  |  |  |  |
| Lung density (HU) | -751 [-814;-668] | -760 [-817;-670] | -750 [-813;-667] | 0.590 |
| Lung gas volume (L) | 0.95 [0.71;1.29] | 0.81 [0.59;1.15] | 1.03 [0.79;1.35] | <0.001 |
| Lung mass (kg) | 0.32 [0.26;0.40] | 0.28 [0.23;0.33] | 0.34 [0.28;0.44] | <0.001 |
| Total injury, fraction | 0.33 [0.20;0.52] | 0.33 [0.19;0.51] | 0.35 [0.20;0.53] | 0.328 |
| GGO, fraction | 0.29 [0.18;0.45] | 0.28 [0.17;0.44] | 0.29 [0.18;0.45] | 0.279 |
| Consolidation, fraction | 0.03 [0.02;0.06] | 0.03 [0.02;0.06] | 0.03 [0.02;0.06] | 0.470 |
|  |  |  |  |  |
| Dorsal |  |  |  |  |
| Lung density (HU) | -651 [-753;-538] | -658 [-768;-546] | -648 [-745;-533] | 0.144 |
| Lung gas volume (L) | 0.78 [0.55;1.14] | 0.69 [0.48;1.05] | 0.85 [0.58;1.22] | <0.001 |
| Lung mass (kg) | 0.43 [0.34;0.54] | 0.36 [0.29;0.44] | 0.46 [0.37; 0.60] | <0.001 |
| Total injury, fraction | 0.55 [0.34;0.73] | 0.53 [0.31;0.72] | 0.56 [0.36;0.75] | 0.072 |
| GGO, fraction | 0.43 [0.28;0.55] | 0.41 [0.25;0.4] | 0.44 [0.30;0.56] | 0.034 |
| Consolidation, fraction | 007 [0.03; 0.15] | 0.07 [0.03;0.14] | 0.07 [0.04;0.17] | 0.263 |
| Dorso-Ventral |  |  |  |  |
| Lung density (HU) | -717 [-782;-635] | -717 [-790;-639] | -716 [-779;-631] | 0.828 |
| Lung gas volume (L) | 0.90 [0.67;1.25] | 0.77 [0.54;1.10] | 0.99 [0.74;1.31] | <0.001 |
| Lung mass (kg) | 0.36 [0.30;0.44] | 0.31 [0.26;0.37] | 0.38 [0.33;0.47] | <0.001 |
| Total injury, fraction | 0.41 [0.25;0.59] | 0.40 [0.23;0.58] | 0.42 [0.25;0.59] | 0.413 |
| GGO, fraction | 0.35 [0.21;0.49] | 0.34 [0.19;0.49] | 0.36 [0.21;0.49] | 0.190 |
| Consolidation, fraction | 0.05 [0.03;0.08] | 0.05 [0.03;0.08] | 0.05 [0.03;0.0.8] | 0.657 |
| Ventral |  |  |  |  |
| Lung density (HU) | -791 [-838;-729] | -790 [-837;-716] | -792 [-839;-735] | 0.161 |
| Lung gas volume (L) | 1.02 [0.77;1.35] | 0.84 [0.62;1.18] | 1.10 [0.86;1.42] | <0.001 |
| Lung mass (kg) | 0.27 [0.22;0.33] | 0.24 [0.20; 0.29] | 0.28 [0.24;0.35] | <0.001 |
| Total injury, fraction | 0.27 [0.16;0.41] | 0.27 [0.15;0.44] | 0.27 [0.16;0.40] | 0.562 |
| GGO, fraction | 0.25 [0.14;0.38] | 0.24 [0.14;0.40] | 0.25 [0.14;0.37] | 0.707 |
| Consolidation, fraction | 0.02 [0.01;0.03] | 0.02 [0.01; 0.04] | 0.02 [0.01;0.03] | 0.024 |
|  |  |  |  |  |
| Peripheral |  |  |  |  |
| Lung density (HU) | -696 [-771;-612] | -690 [-781;-610] | -697 [-767;-631] | 0.984 |
| Lung gas volume (L) | 1.00 [0.75;1.27] | 0.87 [0.64;1.14] | 1.07 [0.83;1.34] | <0.001 |
| Lung mass (kg) | 0.43 [0.33;0.56] | 0.39 [0.30;0.48] | 0.47 [0.34;0.60] | <0.001 |
| Total injury, fraction | 0.47 [0.31;0.61] | 0.46 [0.29;0.61] | 0.47 [0.32;0.60] | 0.754 |
| GGO, fraction | 0.39 [0.27;0.50] | 0.38 [0.25;0.51] | 0.40 [0.27;0.50] | 0.500 |
| Consolidation, fraction | 0.05 [0.03;0.10] | 0.05 [0.03;0.10] | 0.05 [0.03;0.09] | 0.728 |
| Peripheral-Centrohilar |  |  |  |  |
| Lung density (HU) | -729 [-799;-646] | -728 [-803;-655] | -730 [-795;-644] | 0.634 |
| Lung gas volume (L) | 0.73 [0.55;0.97] | 0.63 [0.46;0.84] | 0.79 [0.61;1.02] | <0.001 |
| Lung mass (kg) | 0.27 [0.22;0.35] | 0.24 [0.19;0.29] | 0.29 [0.23;0.38] | <0.001 |
| Total injury, fraction | 0.39 [0.23;0.55] | 0.37 [0.21;0.53] | 0.39 [0.24;0.55] | 0.348 |
| GGO, fraction | 0.33 [0.20;0.45] | 0.32 [0.19;0.45] | 0.33 [0.21;0.46] | 0.225 |
| Consolidation, fraction | 0.04 [0.02;0.08] | 0.04 [0.02;0.09] | 0.04 [0.02;0.08] | 0.722 |
| Centrohilar |  |  |  |  |
| Lung density (HU) | -737 [-803;-644] | -743 [-809;-650] | -731 [-798;-639] | 0.320 |
| Lung gas volume (L) | 0.93 [0.58;1.51] | 0.78 [0.45;1.31] | 1.03 [0.66;1.59] | <0.001 |
| Lung mass (kg) | 0.36 [0.27;0.47] | 0.30 [0.21;0.40] | 0.39 [0.31;0.52] | <0.001 |
| Total injury, fraction | 0.37 [0.21;0.56] | 0.36 [0.19;0.54] | 0.38 [0.22;0.57] | 0.172 |
| GGO, fraction | 0.31 [0.18;0.45] | 0.30 [0.16;0.45] | 0.33 [0.19;0.446] | 0.139 |
| Consolidation, fraction | 0.04 [0.02;0.08] | 0.04 [0.02;0.08] | 0.05 [0.03;0.08] | 0.145 |
| Gradients |  |  |  |  |
| Basal to Apical (HU) | -23 [-49; -0] | -23[-51; -3] | -22 [-49; 1] | 0.424 |
| Dorsal to Ventral (HU) | -61 [-104; -33] | -53 [-94; -24] | -65 [-109; -38] | <0.001 |
| Peripheral to Central (HU) | -12 [-29; 0] | -15 [-32; 0] | -11 [-28; 1] | 0.079 |
| Disease distribution |  |  |  |  |
| Quadrants involvement | None | 215 (25) | 81 (27) | 134 (24) | 0.483 |
| 1 quadrant | 67 (8) | 25 (9) | 42 (8) |
| 2 quadrants | 108 (13) | 30 (10) | 78 (14) |
| 3 quadrants | 96 (11) | 35 (12) | 61 (11) |
| 4 quadrants | 364 (43) | 125 (42) | 239 (43) |
| Bi- or mono-lateral involvement | None | 215 (25) | 81 (27) | 134 (24) | 0.500 |
| mono-lateral | 87 (10) | 27 (9) | 60 (11) |
| Bi-lateral | 548 (65) | 188 (64) | 360 (65) |

Table S3. Quantitative analysis of lung CT images stratified by sex. Continuous data are presented as mean ± SD or median [IQR], as appropriate; categorical variables are reported as count (proportion)). P for T-test, Mann–Whitney Test or Chi square test, as appropriate. List of abbreviations: GGO: ground-glass opacities.

| Variable | OR | 95% CI | P value |
| --- | --- | --- | --- |
| Age (1 year increase) | 1.08 | 1.06-1.10 | < 0.001 |
| Any comorbidities (Ref. No) | 1.65 | 0.93-2.91 | 0.084 |
| Composite SOFA score 1 (Ref. 0) ≥ 2 (Ref. 0) | 2.61 3.14 | 1.56-4.37 1.71-5.74 | < 0.001 < 0.001 |
| PaO2/FiO2, (1 mmHg) | 0.99 | 0.98-0.99 | < 0.001 |
| White Blood Cell (n*103) | 0.97 | 0.92-1.03 | 0.301 |
| Positive Pressure Ventilation (Ref. No) | 2.54 | 0.69-9.34 | 0.160 |
| Global Superimposed Pressure (1 cmH2O) | 1.20 | 1.06-1.35 | 0.003 |
| AIC | 516 |
| BIC | 556 |

Table S4. Multivariable analysis of factors independently associated with probability of mortality including global superimposed pressure in the overall population. N=614. List of abbreviation: AIC: Akaike Information Criterion; BIC: Bayesian Information Criterion; PaO₂ /FiO₂: ratio of arterial oxygen partial pressure to fraction of inspired oxygen; SOFA: Sequential Organ Failure Assessment.

| Variable | OR | 95% CI | P value |
| --- | --- | --- | --- |
| Age, (1 year increase) | 1.08 | 1.06-1.10 | < 0.001 |
| Any comorbidities (Ref. No) | 1.74 | 0.99-3.07 | 0.054 |
| Composite SOFA score 1 (Ref. 0) ≥ 2 (Ref. 0) | 2.18 2.52 | 1.30-3.66 1.36-4.65 | 0.003 0.003 |
| PaO2/FiO2 (1 mmHg) | 0.99 | 0.98-0.99 | < 0.001 |
| White Blood Cell (n*103) | 0.98 | 0.93-1.04 | 0.544 |
| Positive Pressure Ventilation (Ref. No) | 2.93 | 0.80-10.72 | 0.104 |
| Sex (ref. female sex) | 1.87 | 1.13-3.09 | 0.014 |
| AIC | 519 |
| BIC | 559 |

Table S5. Multivariable analysis of factors independently associated with probability of mortality including male sex in the overall population. N=614. Positive pressure ventilation refers to non-invasive respiratory support (e.g., CPAP or BiPAP) applied prior to CT acquisition. List of abbreviation: AIC: Akaike Information Criterion; BIC: Bayesian Information Criterion; PaO₂ /FiO₂: ratio of arterial oxygen partial pressure to fraction of inspired oxygen; SOFA: Sequential Organ Failure Assessment.

| Variable | OR | 95% CI | P value |
| --- | --- | --- | --- |
| Age (1 year increase) | 1.08 | 1.06-1.11 | < 0.001 |
| Any comorbidities (Ref. No) | 1.65 | 0.93-2.93 | 0.085 |
| Composite SOFA score 1 (ref. 0) ≥ 2 (ref. 0) | 2.32 2.67 | 1.37-3.93 1.43-4.98 | 0.002 0.002 |
| PaO2/FiO2 (1 mmHg) | 0.99 | 0.98-0.99 | < 0.001 |
| White Blood Cell (n*103) | 0.97 | 0.92-1.03 | 0.349 |
| Positive Pressure Ventilation (Ref. No) | 2.52 | 0.68-9.36 | 0.168 |
| Global Superimposed Pressure (1 cmH2O) | 1.18 | 1.05-1.34 | 0.005 |
| Sex (ref. female sex) | 1.76 | 1.06-2.92 | 0.028 |
| AIC | 513 |
| BIC | 557 |

Table S6. Multivariable analysis of factors independently associated with probability of mortality including global superimposed pressure and male sex in the overall population. N=614. Positive pressure ventilation refers to non-invasive respiratory support (e.g., CPAP or BiPAP) applied prior to CT acquisition. List of abbreviation: AIC: Akaike Information Criterion; BIC: Bayesian Information Criterion; PaO₂ /FiO₂: ratio of arterial oxygen partial pressure to fraction of inspired oxygen; SOFA: Sequential Organ Failure Assessment.

| Variable | OR | 95% CI | P value |
| --- | --- | --- | --- |
| Age (1 year increase) | 1.08 | 1.06-1.11 | < 0.001 |
| Any comorbidities (Ref. No) | 1.62 | 0.91-2.90 | 0.097 |
| Composite SOFA score 1 (Ref. 0) ≥ 2 (Ref. 0) | 2.28 2.69 | 1.35-3.87 1.44-5.02 | 0.002 0.002 |
| PaO2/FiO2 (1 mmHg) | 0.99 | 0.98-0.99 | < 0.001 |
| White Blood Cell (n*103) | 0.98 | 0.92-1.03 | 0.431 |
| Positive Pressure Ventilation (Ref. No) | 2.59 | 0.67-9.98 | 0.165 |
| Global Superimposed Pressure (1 cmH2O) | 1.04 | 0.84-1.28 | 0.744 |
| Sex (ref. female sex) | 0.55 | 0.11-2.71 | 0.463 |
| Sex (ref. female sex)*Global Superimposed Pressure (1 cmH2O) | 1.20 | 0.94-1.53 | 0.135 |
| AIC | 513 |
| BIC | 562 |

Table S7. Multivariable analysis of factors independently associated with probability of mortality including the interaction between male sex and global superimposed pressure in the overall population. N=614. Positive pressure ventilation refers to non-invasive respiratory support (e.g., CPAP or BiPAP) applied prior to CT acquisition. List of abbreviation: AIC: Akaike Information Criterion; BIC: Bayesian Information Criterion; PaO₂ /FiO₂: ratio of arterial oxygen partial pressure to fraction of inspired oxygen; SOFA: Sequential Organ Failure Assessment.

| Variable | OR | 95% CI | P value |
| --- | --- | --- | --- |
| Age, (1 year increase) | 1.08 | 1.06-1.10 | < 0.001 |
| Any comorbidities (Ref. No) | 1.64 | 0.93-2.90 | 0.088 |
| Composite SOFA score 1 (Ref. 0) ≥ 2 (Ref. 0) | 2.60 3.12 | 1.55-4.35 1.70-5.72 | < 0.001 < 0.001 |
| PaO2/FiO2 (1 mmHg) | 0.99 | 0.98-0.99 | < 0.001 |
| White Blood Cell (n*103) | 0.97 | 0.92-1.03 | 0.310 |
| Positive Pressure Ventilation (Ref. No) | 2.53 | 0.69-9.32 | 0.163 |
| Global Superimposed Pressure (1 cmH2O) | 1.19 | 1.06-1.34 | 0.004 |
| Lung height (1 cm) | 1.01 | 0.91-1.21 | 0.796 |

Table S8. Multivariable analysis of factors independently associated with probability of mortality including global superimposed pressure and lung height in the overall population. N=614. Positive pressure ventilation refers to non-invasive respiratory support (e.g., CPAP or BiPAP) applied prior to CT acquisition. All VIF < 5, mean VIF 1.29. List of abbreviation: PaO₂ /FiO₂: ratio of arterial oxygen partial pressure to fraction of inspired oxygen; SOFA: Sequential Organ Failure Assessment.

| Variable | OR | 95% CI | P value |
| --- | --- | --- | --- |
| Age (1 year increase) | 1.08 | 1.06-1.11 | < 0.001 |
| Any comorbidities (Ref. No) | 1.69 | 0.95-3.00 | 0.073 |
| Composite SOFA score 1 (Ref. 0) ≥ 2 (Ref. 0) | 2.31 2.62 | 1.36-3.91 1.41-4.89 | 0.002 0.002 |
| PaO2/FiO2 (1 mmHg) | 0.99 | 0.98-0.99 | < 0.001 |
| White Blood Cell (n*103) | 0.97 | 0.92-1.03 | 0.321 |
| Positive Pressure Ventilation (Ref. No) | 2.57 | 0.65-9.52 | 0.157 |
| Global Superimposed Pressure (1 cmH2O) | 1.19 | 1.06-1.35 | 0.004 |
| Sex (Ref. Female) | 2.00 | 1.12-3.55 | 0.019 |
| Lung height (1 cm) | 0.95 | 0.84-1.0.6 | 0.369 |

Table S9. Multivariable analysis of factors independently associated with probability of mortality including global superimposed pressure, male sex, and lung height in the overall population. N=614. Positive pressure ventilation refers to non-invasive respiratory support (e.g., CPAP or BiPAP) applied prior to CT acquisition. All VIF < 5, mean VIF 1.35. List of abbreviation: PaO₂ /FiO₂: ratio of arterial oxygen partial pressure to fraction of inspired oxygen; SOFA: Sequential Organ Failure Assessment.
